# Differences in Food Consumption and Meal Patterns in Texas School Children by Grade

**Published:** 2007-03-15

**Authors:** Adriana Pérez, Deanna M Hoelscher, Henry Shelton Brown, Steven H Kelder

**Affiliations:** School of Public Health, Division of Biostatistics. All authors are also affiliated with the Hispanic Health Research Center at the Lower Rio Grande Valley, Brownsville, Tex; The University of Texas Health Science Center at Houston, Division of Health Promotion/Behavioral Sciences and Human Nutrition Center, Houston, Tex. All authors are also affiliated with the Hispanic Health Research Center at the Lower Rio Grande Valley, Brownsville, Tex; The University of Texas Health Science Center at Houston, School of Public Health, Division of Management Policy and Community Health, Brownsville, Tex. All authors are also affiliated with the Hispanic Health Research Center at the Lower Rio Grande Valley, Brownsville, Tex; The University of Texas Health Science Center at Houston, Center for Health Promotion and Prevention Research, Houston, Tex. All authors are also affiliated with the Hispanic Health Research Center at the Lower Rio Grande Valley, Brownsville, Tex

## Abstract

**Introduction:**

Having information about dietary patterns at different ages and stages in children's physical development is important in developing nutritional interventions. The purpose of this study was to examine differences in food choices between 4th-, 8th-, and 11th-grade students. The results provide information that can be used to tailor behavioral-based nutritional programs for children.

**Methods:**

We determined food consumption patterns using validated data from the School Physical Activity and Nutrition survey; the survey is used as part of a surveillance program of public school students conducted by the University of Texas Health Science Center at Houston in partnership with the Texas Department of State Health Services. The sample included a total of 15,173 children in grades 4 (6235), 8 (5362), and 11 (3576). Multistage probability sampling weights were used. Odds ratios were computed controlling for sex, body mass index, and race and ethnicity, and cross-sectional patterns were determined using multivariate logistic regression.

**Results:**

Children in grades 8 and 11 were more likely to consume hamburger and other meats, cheese, breads, buns, and rolls, and sweet rolls compared with 4th-grade students. In contrast, 4th-grade students were more likely to consume peanuts or peanut butter, yogurt, cereal, fruit, and milk compared with 8th- and 11th-grade students. Eighth- and eleventh-grade students were more likely to consume snacks than 4th-grade students.

**Conclusion:**

Using cross-sectional data to assess differences in dietary intake and meal patterns by grade can provide readily accessible information to develop a needs assessment or intervention materials for children and adolescents. Different intervention development approaches are necessary among children in different grades.

## Introduction

During the past two decades, the prevalence of overweight in children in the United States has significantly increased. These increases are especially high among children from African American and Latino and Hispanic populations (1). Children and adolescents in Texas have rates of overweight that are higher than national estimates (2). Interventions to address child and adolescent overweight should emphasize both dietary intake and physical activity (3,4).

Behavioral interventions that address dietary intake changes among youth usually target specific eating patterns or foods rather than nutrients (4,5). Developing nutrition interventions for children involves considering food preferences and dietary patterns that may change over time. Data from two longitudinal studies indicate that as children become older, their eating patterns change significantly; changes include decreased consumption of fruits, vegetables, desserts, candy, mixed meats, and milk and increased consumption of soft drinks, sweetened beverages, salty snacks, poultry, seafood, and beef (6,7).

Dietary changes are affected by children's developmental levels, accessibility to foods in different grade levels, and the degree to which parents or other adults serve as gatekeepers to food. Many studies have examined differences in food patterns and dietary patterns using serial cross-sectional surveys of dietary intake (8,9) or longitudinal samples of dietary intake over time (6,7). Other studies have analyzed nutrients, but not foods, over time (10,11).

A cross-sectional comparison of three grades has a key advantage over comparisons derived from longitudinal or cohort studies. Longitudinal studies can be used to determine changes in food and meal patterns of students as they grow older and progress to higher grade levels. However, because cohort data are collected over an extended period of time, secular influences that vary by age can confound the comparison. Examining group differences among grades at one point in time eliminates differences caused by secular trends over time, and cross-sectional data can be collected quickly and provide readily accessible point estimates. Although a few studies do exist, there is a paucity of data on the comparison of food choices among children of different developmental ages and grade levels, especially in an ethnically diverse population with a significant number of Latino and Hispanic children.

The purpose of this study was to examine cross-sectional differences in food choices between 4th-, 8th-, and 11th-grade students in Texas. We hypothesized that analysis of food consumption among three age groups of children would result in different patterns, including 1) a linear pattern in which consumption increases or decreases systematically from 4th to 8th to 11th grade; or 2) a quadratic pattern in which consumption changes among 8th-grade students compared with the 4th-grade students and then later changes in the opposite direction among 11th-grade students compared with 8th-grade students. Knowledge of these patterns and their underlying causes can provide insight into the determinants that influence food consumption as well as whether the consumption patterns are transitory or stable over time. The determinants influencing food consumption can, in turn, be targeted with intervention strategies. For example, food patterns that increase linearly may be influenced by a student's increasing independence and mobility, while food patterns that follow a quadratic pattern may be related to the developmental stage of the child or the changes in food environment that occur during middle school. We explore both types of relationships in this study.

## Methods

The School Physical Activity and Nutrition (SPAN) project is an obesity monitoring program developed by The University of Texas School of Public Health and conducted by the Texas Department of State Health Services (12). As part of the SPAN project, a survey is used to collect data on students' eating behaviors, nutrition knowledge, and physical activity. SPAN uses a stratified, multistage probability sample of public school children in Texas that includes representative samples of white, black or African American, and Mexican-American-Latino or Hispanic youth. A full description of the SPAN study design and its participants has been previously documented (2), and a brief summary is provided here. The sampling scheme provided regionally representative data for the state on an annual basis: school year 1 (2000-2001) and school year 2 (2001-2002) (2). A single grade was selected to represent each developmental level of school children: 4th grade for elementary school, 8th grade for middle school, and 11th grade for high school. The sample consisted of 6235 children for 4th grade from a grade population of 390,863, 5362 children for 8th grade from a grade population of 288,584, and 3576 children for 11th grade from a grade population of 249,363. The response rates by grade were 76% for 4th grade, 65% for 8th grade, and 43% for 11th grade. Approval for the SPAN study was obtained from the Committee for the Protection of Human Subjects at the University of Texas Health Science Center at Houston as well as the Texas Department of State Health Services Institutional Review Board and participating school districts. Parents completed either an active or passive consent (depending on school district procedures for parental consent), and children completed a child assent form.

Two versions of the SPAN food frequency questionnaire, one for elementary school students and another for middle and high school students, were previously developed and evaluated for psychometric properties for an ethnically diverse population (13-15). A brief summary of the validation results is included here. Validity of the food consumption items in each version was assessed using 24-hour recalls of food consumption (a single recall was used for the elementary school version). Agreement measures showed fair to good validity in the version for the elementary school students (13-15) and showed moderate to good validity in the version for the middle and high school students (14). SPAN surveys were administered using standard protocols and included items on dietary intake, physical activity, meal patterns, and nutrition knowledge; children's height and weight measurements were also recorded. Body mass index (BMI) was calculated using the standard equation of weight in kilograms divided by the square of the height in meters. Children were classified as underweight/normal weight (<85th percentile), at risk of overweight (≥85th percentile to <95th percentile) and overweight (≥95th percentile) using the Centers for Disease Control and Prevention's (CDC's) BMI-for-age growth charts for girls and boys (www.cdc.gov/growthcharts/).

Items on food and meal choice behaviors and vitamin consumption from the SPAN questionnaires were used for this analysis. Food choice questions included 20 categories of food, ranging from gravy to breaded and fried meats to fruits and vegetables. Questions were developed to target marker foods for nutrients or groups of interest, such as high-fat foods, fruits and vegetables, and dairy foods. Questionnaires were administered Tuesday through Friday, so that data on weekday food consumption could be captured.

Food consumption was assessed by asking about the frequency of intake of the food or foods on the previous day (e.g., "Yesterday, did you eat fruit?"). Response categories for each food item included 1) none/I did not eat that food, 2) I ate the food one time, 3) I ate the food two times, and 4) I ate the food three or more times. Responses for one, two, and three or more times were combined into one category to indicate any consumption of that food on the previous day, and responses for "none/I did not eat that food" were coded as no consumption of that food during the previous day. Beverage consumption was assessed using items about sodas, any kind of milk, fruit juice, and fruit-flavored drinks; these items were coded in the same manner as the food choice questions.

Meal pattern behaviors included questions about the previous day's behaviors, including whether or not breakfast was eaten, the number of meals eaten per day, and whether or not a snack was eaten; these questions were worded slightly differently for the elementary and secondary grade surveys. For 4th-grade students, the question about breakfast consumption was, "Yesterday, did you eat breakfast?" compared with, "Do you usually eat or drink something for breakfast?" for 8th- and 11th-grade students. For the 8th- and 11th-grade students, responses of "almost always," "always," or "sometimes" were recorded as a yes response, and "almost never" or "never" were recorded as a no response. The question about vitamin consumption assessed supplement intake (not specified) on the previous day, with a yes/no format. Vitamin consumption was determined by asking 4th-grade students the question, "Yesterday, did you take a vitamin pill?" For 8th- and 11th-grade students vitamin consumption was determined by asking the question, "Do you usually take a vitamin or mineral pill?"

All estimates and statistical tests accounted for sample design features. STATA (version 7.0, StataCorp LP, College Station, Tex) was used to analyze the data. Weighted means, standard deviations, and proportions for demographic characteristics were computed; the Pearson chi-square test was used to evaluate demographic differences by grade. Adjusted odds ratios (AORs) for food choice behavior or meal choice behavior associated with student grade and 95% confidence intervals (95% CIs) were calculated using sampling-weight logistic regression models for each food item, using 4th-grade students as the referent group. Adjustment variables were sex, race and ethnicity, and BMI. We assessed cross-sectional linear and quadratic differences among grades for each food item by treating categorical variables as continuous variables and by using coefficients for orthogonal polynomials with multiple logistic regression models. We wanted to determine whether the relationship between grade level and the logit of consuming a food item had significant linear components, quadratic components, or both. To measure the linear component, we coded categorical variables as −1 for 4th grade, 0 for 8th grade, and +1 for 11th grade. To measure the quadratic component, we coded 1 for 4th grade, −2 for 8th grade, and 1 for 11th grade. An α level of .05 was established a priori as the probability of incurring a type I error.

## Results

The mean ages of the students were 9.7 years for 4th grade, 13.7 years for 8th grade, and 16.7 years for 11th grade (Table 1). Fifty-four percent of the children were male; no statistically significant differences were found in the sex distribution by grade (*P* = .62). The distribution of race and ethnicity by grade differed significantly (*P* = .04) and was consistent with state population proportions (Table 1). Overall, 20% of the student population surveyed was classified as overweight, and the distribution of BMI means and categories was significantly different among grades (*P* < .01).

Table 2 shows percentages of foods and beverages consumed by grade level. During the previous day, 60% or more of school children in 4th grade ate the following: cheese; breads, buns, bagels, tortillas or rolls; vegetables; French fries or chips; and fruit. Also, 60% or more drank any kind of milk; fruit juice; and any soda or soft drink. In 8th grade, two thirds of students ate hamburger meat, hot dogs, sausage, steak, bacon, or ribs (66%); 77% ate cheese; 84% ate breads, buns, bagels, tortillas or rolls; 74% ate French fries or chips; and more than 75% drank any soda or soft drink. Foods most frequently eaten in 11th grade included hamburger meat, hot dogs, sausage, steak, bacon, or ribs (67%); cheese (75%); breads, buns, bagels, tortillas, or rolls (86%); French fries or chips (66%); and vegetables (61%). Sodas or soft drinks were the most frequently recorded beverage choice among 11th-grade school children in Texas. More than 80% of 11th-grade students ate breakfast, more than 88% had more than one meal, more than 85% consumed at least one snack, and 24% reported consuming vitamin supplements (Table 3).

Odds ratios adjusted by sex, race and ethnicity, and BMI for consumption of each food group by grade are presented in Table 4 along with *P* values for the linear and quadratic analyses. Although these data are cross-sectional, we examined whether changes in food intake increased linearly across grade levels (i.e., differences in consumption between 4th and 8th grades were the same as differences in consumption between 8th and 11th grades) or did not increase linearly across grade levels (i.e., quadratic). Statistically significant differences in food choice behaviors were evident among 4th-, 8th-, and 11th-grade students. Students in 8th and 11th grade were almost twice as likely as 4th-grade students to consume hamburger meat, hot dogs, sausage, steak, bacon, or ribs as well as cheese. Students in 8th grade were almost twice as likely to consume French fries or chips as 4th-grade students (AOR = 1.96; 95% CI, 1.46–2.63). Compared with 4th grade students, children in both higher grades reported higher (increasing quadratic) consumption of hamburger meats, hot dogs, sausage, steak, bacon, or ribs; cheese; breads, buns, bagels, tortillas, or rolls; sweet rolls, doughnuts, cookies, brownies, pies, or cakes; and any soda or soft drink. This pattern indicates a polynomial choice behavior for these food items. Children in higher grades reported less consumption of peanuts or peanut butter, yogurt, cereal, fruit, and milk than elementary school children. For meal patterns, the only significant difference across grade levels was snack consumption (Table 5). This difference increased linearly across grade levels (*P* <.001), indicating that students in the two upper-level grades consumed more snacks than students in the 4th grade.

## Discussion

Our results show that children's diets are marked by greater consumption of less healthful foods in higher grades compared with lower grades; we found a higher self-reported intake of higher-fat foods (e.g., high-fat meats, sweet rolls, French fries) and soft drinks among children in higher grades compared with children in lower grades. In addition, a lower consumption of healthier foods (e.g., fruit, yogurt, milk, cereal, peanuts and peanut butter) was reported among students in 8th and 11th grades compared with students in 4th grade. No significant differences were reported by grade for consumption of fried meat, chicken, and fish or vegetables, and there were no significant differences reported across grades for breakfast consumption, number of meals consumed, or vitamin pill intake. Children in secondary schools were more likely to report consumption of snacks compared with children in elementary school.

Our results are consistent with other studies that show differences in eating patterns across grades or age groups for children. In the Child and Adolescent Trial for Cardiovascular Health (CATCH) cohort, children reported a linear decrease in consumption of breakfast, fruits, and vegetables from elementary to secondary grades (6,7). Data from the Bogalusa cohort show a statistically significant linear decrease in consumption of fruits and juice, mixed meats, desserts, candy, and milk in young adulthood (ages 19-28 years) compared with children aged 10 years (7). Although SPAN data indicated a slight decrease in vegetable consumption among 8th-grade students compared with 4th-grade students, the difference was not statistically significant.

Consumption of more healthy foods among elementary school students compared with students in secondary school (linear or quadratic) could be caused by the school meal environment (e.g., presence of fast food and á la carte lines), greater parental monitoring and control of food intake, social norms about food intake, and the increased off-campus mobility and independence of middle and high school students. Additionally, in elementary school, parental concerns may be more focused on nutrition and physical activity; however, as children grow and physically develop, other health-related issues such as substance abuse, alcohol consumption, and sexual behaviors may increase in frequency and importance (16,17) and may tend to take precedence over nutrition and physical activity (16,17).

When children enter middle school, the school meal environment usually changes. Generally, elementary school meals follow the U.S. Department of Agriculture reimbursable school meal pattern, along with a few á la carte snacks (18). Middle schools and high schools often serve food in an all-you-can-eat style or include multiple options. Further, older students are more likely to have extra money to use in vending machines. Therefore, it would be expected that food consumption patterns would vary across grades (18,19). The results of this study indicate that many middle school students do consume foods such as hamburgers and French fries on a daily basis. In fact, based on the results of the quadratic analyses, it appears that the increased availability of these foods (e.g., hamburger meat, sodas) in middle schools leads to increased consumption among middle school students; consumption tapers off among high school students. The increased availability of novelty foods among middle school students — who are establishing their own identities and trying new behaviors — may lead to an increased consumption of these foods. They are no longer a novelty among high school students — who are more mature and emotionally developed than middle school students — and this may explain the decreased consumption among this age group. Environmental interventions, such as the current Texas Public School Nutrition Policy (20), that seek to limit availability of these foods for all grades should be encouraged.

Recently, it has been hypothesized that the strategic placement of fast food restaurants and convenience stores near schools adversely affects food consumption patterns of children (21). High school is also a time of increased autonomy; students are driving and eating out without their family's influence. In addition, the increase in the number of snacks across grade levels may indicate increased mobility, less traditional meal times, and increased access to food and money to buy food (6). Thus, interventions that target high school students should focus on peer influences and strategies for eating out or planning menus so that students learn to make healthy food choices outside of the school environment.

In our study, there were no significant differences in breakfast consumption across grade levels, as noted in other studies (6,22-24). This finding may be an artifact of the difference in questionnaire items by grade. The 4th-grade survey assessed breakfast consumption on the previous day; the 8th- and 11th-grade survey assessed usual breakfast consumption. In addition, the population in the current study was more ethnically diverse than populations in previous reports. Cereal consumption did decrease among children in higher grades compared with 4th-grade children, and cereal is often consumed for breakfast.

From these data, it appears that elementary students are more likely to consume more healthful foods than middle and high school students; however, results from SPAN also indicate that 4th-grade students were more likely to be overweight than 8th- or 11th-grade students (2). The data for our analyses were adjusted for BMI, race and ethnicity, and sex to account for the effect of different body proportions at each grade level. In addition, even though frequency of food consumption is assessed in the SPAN questionnaire, amounts or portion sizes of foods are not, so it may be that 4th-grade students are consuming larger portions than necessary and ingesting more calories, even if they consume a healthier diet overall. Further, it may be that the increased prevalence of overweight among elementary students may result from decreased physical activity rather than excess caloric consumption. Thus, it is imperative to continue to provide empirically tested intervention programs in elementary schools, especially interventions that target the family as well as the child.

There were several limitations to our study. First, only one day of diet was measured by the questionnaire items (i.e., "yesterday" consumption patterns), and one day is not appropriate for capturing individual dietary variation; however, we used the food variable as a group-level analysis, which is the most appropriate and representative analysis for this population. Second, the SPAN questionnaire recorded weekday intake solely because of cost considerations, and data derived from the study are intended to provide information to evaluate school-based interventions and school-day eating patterns. Third, estimates of intake from the SPAN questionnaires are not as accurate as 24-hour dietary recalls (25). Additionally, they do not measure individual-level intake; our instruments were developed as a population-level screening tool, useful for epidemiologic studies as a group-level estimator of foods commonly eaten by children. Furthermore, as a population-level tool, the interpretation of SPAN data is appropriate as a method for ranking the food intake of a population, discriminating among individuals or populations with regard to higher food intake compared with lower food intake, as well as examining interrelationships between diet and other variables. Fourth, self-report recall bias could potentially influence the results, although we do not expect recall bias to vary significantly across 4th, 8th, or 11th grades, because validity and reliability data were similar for both the elementary and secondary level questionnaires. Fifth, although consumption frequency was measured, food portion sizes were not, and this factor could affect the relative outcome of our analysis. Finally, there were slight differences in the wording of some of the questions between the questionnaire for elementary school students and the questionnaire for middle and high school students as described in the methods. The changes were based on developmental work previously done on the questionnaire to ensure better understanding by elementary school children, who may have limited reading skills.

The strengths of our study include the sampling design, which accounts for ethnic diversity in Texas; a large representative sample; and the validity and reliability of the questionnaires used (13-15). The SPAN sample also included a large percentage of African American and Latino and Hispanic students.

Significant differences in food behaviors by grade level were found in an ethnically diverse cross-sectional sample of school children in Texas. Data from this study indicate that school-based interventions should be targeted to the age, grade, developmental level, and food environment of the child. Future research should focus on factors that cause differences by age or grade level, as well as which age groups can be considered representative for survey research.

Texas legislation mandates that elementary schools implement a school-based health promotion program by school year 2007–2008 (i.e., Bienestar, CATCH, Planet Health) that focuses on nutritional education with family components (26). A recent review of prevention programs for child overweight found that, although fewer intervention programs for secondary students were conducted, those programs seemed to be more effective at obesity prevention than some of the elementary school programs (27). These results also indicate the need for legislative efforts targeting secondary schools that are similar to those implemented for elementary schools in Texas.

The results of this study provide implications for tailoring and focusing behavioral-based nutritional intervention programs, which may include the linear and quadratic components reported. For 4th-grade students, intervention programs should focus on maintaining consumption of foods that are currently eaten and avoiding foods (e.g., á la carte foods, fast foods) that students will be exposed to in increasing amounts in middle and high school. Although 4th-grade students seemed to have a healthier diet pattern overall compared with older students, improvements can be made. For example, only 61% of the 4th-grade students ate any vegetables (not including French fries or chips), and only 71% ate fruit. These percentages indicate that students are consuming fewer fruits and vegetables than what is recommended for this age group in the 2005 U.S. Dietary Guidelines (www.health.gov/dietaryguidelines/dga2005/document/) and the MyPyramid Plan (www.mypyramid.gov/). Interventions for these students should stress the nutritional importance and social aspects of healthy eating, as well as appropriate food choices for both children and their parents.

For 8th- and 11th-grade students, programs should focus on strategies for coping with changes in the cafeteria environment, including the availability of certain foods such as French fries. Results from the quadratic analysis indicate that middle school students might need these strategies more than high school students, or alternatively, they might need healthy-tasting foods that satisfy the developmental need for novelty, such as ethnic meals. During high school, interventions should aim to make healthful foods (e.g., fruit, yogurt) available at places that students frequent both in and out of school. Such interventions must address the issue of mobility. In addition, behavioral strategies that emphasize peer influences and food selection for an increasingly independent population should be implemented. Similarly, 8th-grade students showed an increased access to sodas and soft drinks in middle school and were more likely to report drinking these beverages. Children in the 11th grade were less likely to drink sodas and soft drinks than children in 8th grade; this difference may be because the quadratic increase is temporary, compared with a linear trend. This finding does not indicate that high school is a less important place to intervene, but given the evidence for behavioral and physiological tracking of maintenance of behavioral patterns over time (28,29), early and continued intervention should improve the expected long-term consumption pattern.

## Figures and Tables

**Table 1:** Demographic Characteristics of Texas School Children, by Grade^a^, School Physical Activity and Nutrition (SPAN) Monitoring System, 2000–2001 and 2001–2002

Characteristic	4th Grade (n = 6235)	8th Grade (n = 5362)	11th Grade (n = 3576)	Total (n = 15,173)	*P*
Age, y, mean (SE)	9.7 (0.03)	13.7 (0.03)	16.7 (0.05)	13.1 (0.30)	<.001
Sex, %					
Male	51.9	54.6	55.0	53.7	.62
Female	48.1	45.4	45.0	46.3	
Race and ethnicity, %					
Black or African American	11.8	11.2	9.3	10.9	.04
Mexican-American, Latino, or Hispanic	45.1	40.9	26.7	38.3	
White or other race or ethnicity^b^	43.1	47.9	64.0	50.9	
BMI, mean (SE)	20.2 (0.23)	22.9 (0.24)	24.1 (0.26)	22.3 (0.18)	<.001
BMI categories^c^, %					
Underweight/normal weight	56.2	63.5	68.8	62.4	.01
At risk of overweight	18.2	17.8	16.8	17.6	
Overweight	25.6	18.8	14.5	20.0	

BMI indicates body mass index.

a Percentages are based on population sizes estimated using sampling weights: 4th grade, 390,863; 8th grade, 288,584; and 11th grade 847,810.

b Includes white-non-Hispanic-non-Latino, Asian, American Indian or Alaska Native, and Native Hawaiian or other Pacific Islander.

c Children were classified as underweight/normal weight (<85^th^ percentile), at risk of overweight (≥85^th^ percentile to <95^th^ percentile), and overweight (≥95^th^ percentile), using the Centers for Disease Control and Prevention's BMI-for-age growth charts for girls and boys.

**Table 2:** Survey Results for Food Choice Behaviors of Texas School Children, by Grade^a^, School Physical Activity and Nutrition (SPAN) Monitoring System, 2000–2001 and 2001–2002

Question: Yesterday, did you eat/drink . . .	4th Grade, % (95% CI)	8th Grade, % (95% CI)	11th Grade, % (95% CI)	Total, % (95% CI)
Hamburger meat, hot dogs, sausage (chorizo), steak, bacon, or ribs?	49.3 (44.6-54.0)	65.6 (60.1-70.7)	67.4 (64.2-70.4)	60.2 (57.0-63.2)
Any fried meat with a crust, like fried chicken, chicken nuggets, chicken fried steak, fried pork chops, or fried fish?	41.2 (36.0-46.5)	37.9 (33.1-43.0)	42.9 (37.4-48.5)	40.6 (37.6-43.6)
Gravy (either on a food or by itself)?	13.8 (11.2-16.9)	14.5 (11.4-18.3)	18.8 (14.2-24.4)	15.5 (13.6-17.6)
Peanuts or peanut butter?	25.1 (22.9-27.6)	17.7 (15.1-20.7)	19.1 (14.7-24.4)	20.9 (18.8-23.1)
Cheese by itself or on your food?	61.3 (58.2-64.3)	77.3 (74.2-80.2)	74.8 (67.9-80.6)	70.7 (67.6-73.6)
Any kind of milk?	85.3 (83.5-87.0)	72.2 (69.7-74.5)	62.0 (56.8-67.0)	74.0 (71.0-76.8)
Yogurt or cottage cheese or drink a yogurt drink?	20.8 (17.0-25.1)	11.1(8.1-15.0)	11.2(7.6-16.4)	14.7 (12.5-17.2)
Rice, macaroni, spaghetti, or pasta noodles?	42.9 (39.9-46.0)	37.6 (34.2-41.1)	42.6 (38.0-47.4)	41.0 (38.7-43.4)
Any bread, bun, bagel, tortilla, or roll?	72.4 (69.0-75.5)	83.5 (81.7-85.2)	85.8 (81.6-89.2)	80.1 (78.2-81.9)
Any hot or cold cereal?	48.5 (45.2-51.9)	36.7 (33.3-40.2)	29.5 (24.4-35.3)	38.8 (35.9-41.9)
French fries or chips?	60.3 (55.2-65.1)	74.2 (70.4-77.7)	65.5 (51.1-77.5)	66.5 (61.7-71.0)
Any vegetables?	61.0 (58.2-63.8)	55.1 (49.1-60.9)	61.3 (55.9-66.4)	59.1 (56.3-61.8)
Beans such as pinto beans, baked beans, kidney beans, refried beans, or pork and beans?	18.5 (15.8-21.4)	22.0 (18.9-25.5)	24.1 (20.5-28.0)	21.3 (19.4-23.4)
Fruit?	71.5 (67.3-75.4)	51.5 (47.6-55.5)	46.8 (39.4-54.4)	57.4 (53.7-61.0)
Fruit juice?	60.9 (55.8-65.7)	52.6 (46.9-58.3)	49.2 (39.8-58.8)	54.7 (50.6-58.6)
Any fruit-flavored drink?	57.9 (55.3-60.4)	51.7 (45.7-57.7)	50.6 (46.6-54.6)	53.7 (51.0-56.3)
Any soda or soft drink?	61.2 (56.3-65.8)	75.7 (73.5-77.8)	70.6 (66.6-74.2)	68.8 (66.2-71.2)
A frozen dessert?	40.6 (37.6-43.7)	35.6 (32.7-38.5)	24.3 (20.0-29.2)	34.1 (31.5-36.8)
Sweet rolls, doughnuts, cookies, brownies, pies, or cake?	33.5 (29.9-37.3)	45.9 (41.1-50.7)	45.0 (40.7-49.3)	41.1 (38.2-44.0)
Any chocolate candy?	30.4 (27.1-33.9)	35.0 (29.7-40.8)	30.6 (27.2-34.4)	32.1 (29.6-34.6)

CI indicates confidence interval.

a Percentages are weighted and indicate the proportion of participant responses categorized as yes.

**Table 3:** Survey Results for Meal Choice and Vitamin Pill Behaviors of Texas School Children, by Grade^a^, School Physical Activity and Nutrition (SPAN) Monitoring System, 2000–2001 and 2001–2002

Question: Yesterday, did you . . . b	4th Grade,% (95% CI)	8th Grade,% (95% CI)	11th Grade,% (95% CI)	Total,% (95% CI)
Eat breakfast?	81.8 (78.7-84.6)	81.1 (77.6-84.1)	80.5 (75.8-84.4)	81.2 (79.0-83.2)
Have more than one meal?	86.8 (84.0-89.1)	87.6 (84.6-90.1)	88.5 (85.7-90.7)	87.6 (86.0-89.0)
Have a snack?	76.8 (72.9-80.3)	82.5 (80.5-84.4)	85.5 (81.0-89.1)	81.3 (79.2-83.3)
Take a vitamin pill?	29.0 (25.4-32.9)	27.5 (23.9-31.3)	24.4 (22.1-26.9)	27.1 (25.0-29.3)

CI indicates confidence interval.

a Percentages are weighted and indicate the proportion of participant responses categorized as yes.

b Questions differed slightly among respondents.

**Table 4:** Food Choice Behavior Among Texas School Children, by Grade, School Physical Activity and Nutrition (SPAN) Monitoring System, 2000–2001 and 2001–2002

Question: Yesterday, did you eat/drink . . .	Grade	AOR^a^ (95% CI)	*P* b	*P* c
Hamburger meat, hot dogs, sausage (chorizo), steak, bacon, or ribs?	4th	Ref	<.001	.03
	8th	1.99 (1.47-2.71)		
	11th	2.21 (1.70-2.86)		
Any fried meat with a crust, like fried chicken, chicken nuggets, chicken fried steak, fried pork chops, or fried fish?	4th	Ref	.47	.17
	8th	0.88 (0.65-1.20)		
	11th	1.13 (0.81-1.57)		
Gravy (either on a food or by itself)?	4th	Ref	.05	.55
	8th	1.10 (0.74-1.62)		
	11th	1.50 (0.99-2.27)		
Peanuts or peanut butter?	4th	Ref	.01	.07
	8th	0.65 (0.51-0.83)		
	11th	0.68 (0.53-0.88)		
Cheese by itself or on your food?	4th	Ref	<.001	<.001
	8th	2.20 (1.77-2.74)		
	11th	1.89 (1.34-2.67)		
Any kind of milk?	4th	Ref	<.001	.08
	8th	0.45 (0.37-0.55)		
	11th	0.28 (0.22-0.36)		
Yogurt or cottage cheese or drink a yogurt drink?	4th	Ref	.02	.11
	8th	0.50 (0.32-0.80)		
	11th	0.53 (0.30-0.91)		
Rice, macaroni, spaghetti, or pasta noodles?	4th	Ref	.99	.03
	8th	0.81 (0.66-1.00)		
	11th	1.00 (0.78-1.28)		
Any bread, bun, bagel, tortilla, or roll?	4th	Ref	<.001	.02
	8th	2.08 (1.62-2.66)		
	11th	2.55 (1.81-3.58)		
Any hot or cold cereal?	4th	Ref	<.001	.59
	8th	0.65 (0.53-0.80)		
	11th	0.48 (0.36-0.65)		
French fries or chips?	4th	Ref	.41	.01
	8th	1.96 (1.46-2.63)		
	11th	1.33 (0.67-2.62)		
Any vegetables?	4th	Ref	.40	.08
	8th	0.75 (0.57-0.98)		
	11th	0.90 (0.71-1.15)		
Beans such as pinto beans, baked beans, kidney beans, refried beans, or pork and beans?	4th	Ref	.02	.78
	8th	1.28 (0.97-1.69)		
	11th	1.53 (1.07-2.18)		
Fruit?	4th	Ref	<.001	.01
	8th	0.41 (0.32-0.54)		
	11th	0.34 (0.24-0.49)		
Fruit juice?	4th	Ref	.09	.46
	8th	0.73 (0.53-1.02)		
	11th	0.68 (0.44-1.06)		
Any fruit-flavored drink?	4th	Ref	.13	.33
	8th	0.82 (0.64-1.05)		
	11th	0.85 (0.70-1.05)		
Any soda or soft drink?	4th	Ref	.01	<.001
	8th	1.96 (1.55-2.49)		
	11th	1.48 (1.09-2.02)		
A frozen dessert?	4th	Ref	<.001	.06
	8th	0.89 (0.73-1.08)		
	11th	0.54 (0.41-0.72)		
Sweet rolls, doughnuts, cookies, brownies, pies, or cake?	4th	Ref	<.001	.06
	8th	1.87 (1.46-2.39)		
	11th	1.91 (1.47-2.49)		
Any chocolate candy?	4th	Ref	.24	.11
	8th	1.36 (0.98-1.90)		
	11th	1.16 (0.91-1.47)		

AOR indicates adjusted odds ratio; CI, confidence interval.

a Odds ratio adjusted for sex, race and ethnicity, and body mass index.

b *P* value for linear component.

c *P* value for quadratic component.

**Table 5:** Meal Choice and Vitamin Pill Behaviors Among Texas School Children, by Grade, School Physical Activity and Nutrition (SPAN) Monitoring System, 2000–2001 and 2001–2002

Question: Yesterday, did you . . . a	Grade	AOR^b^ (95% CI)	*P* c	*P* d
Eat breakfast?	4th	Ref	.91	.84
	8th	1.04 (0.80-1.35)		
	11th	1.02 (0.71-1.46)		
Have more than one meal?	4th	Ref	.32	.79
	8th	1.13 (0.80-1.59)		
	11th	1.17 (0.86-1.57)		
Have a snack?	4th	Ref	<.001	.25
	8th	1.59 (1.23-2.06)		
	11th	1.97 (1.33-2.94)		
Take a vitamin pill?	4th	Ref	.19	.43
	8th	1.01 (0.78-1.31)		
	11th	0.86 (0.68-1.08)		

AOR indicates adjusted odds ratio; CI, confidence interval.

a Questions differed slightly among respondents.

b Odds ratio adjusted for sex, race and ethnicity, and body mass index.

c *P* value for linear component.

d *P* value for quadratic component.
